# Impact of the first COVID-19 pandemic wave on the Scottish Multiple Sclerosis Register population

**DOI:** 10.12688/wellcomeopenres.16349.1

**Published:** 2020-11-25

**Authors:** Peter M. Fernandes, Martin O'Neill, Patrick K.A. Kearns, Sinforosa Pizzo, Chrissie Watters, Stuart Baird, Niall J.J. MacDougall, David P.J. Hunt

**Affiliations:** 1 Anne Rowling Clinic, Centre for Clinical Brain Sciences, University of Edinburgh, Edinburgh, UK; 2 Clinical & Protecting Health Directorate, Public Health Scotland, Edinburgh, UK; 3 Institute of Genetics and Molecular Medicine, University of Edinburgh, Edinburgh, UK; 4 Institute of Neurological Sciences, NHS Greater Glasgow and Clyde, Glasgow, UK; 5 UK Dementia Research Institute, University of Edinburgh, Edinburgh, UK

**Keywords:** COVID-19, multiple sclerosis, MS, Scotland, SARS-CoV-2, mortality

## Abstract

**Background**: The impact of the coronavirus disease 2019 (COVID-19) pandemic on people with multiple sclerosis (MS) is a major current concern, in particular the risk of death. Here we describe the impact of the first wave of COVID-19 infections (Mar 2020-July 2020) on the Scottish MS Register (SMSR) population, a cohort of 4702 individuals with MS, all newly diagnosed in the past decade.

**Methods**: We established a clinician alert system, linking the SMSR with the Electronic Communication of Surveillance in Scotland (ECOSS). This allows identification of patients within this cohort who had a positive SARS-CoV-2 PCR test. The SMSR was also linked to death records from National Records Scotland.

**Results**: Of 4702 people with MS, 246 severe acute respiratory syndrome coronavirus 2 (SARS-CoV-2) PCR tests were performed, of which 17 were positive. The proportion of positive tests were similar to the general Scotland population (Observed PCR confirmed cases = 17, expected = 17.5, O/E = 0.97, 95% CI: 0.60 – 1.56, *p*=.90). Between 1 ^st^ March – 31 ^st^ July 2020 12 individuals on the SMSR died, 5 of which were linked to COVID-19 (1 PCR confirmed, 4 clinical diagnoses without PCR confirmation). This number of COVID-19-related deaths was higher than expected (observed deaths = 5, expected deaths = 1.2, O/E = 4.03, 95% CI = 1.48 – 8.94, *p*=.01). All COVID-19-related deaths in the SMSR occurred in individuals with advanced disability (Expanded Disability Status Scale ≥7), and no deaths occurred in patients receiving disease modifying therapy (DMT) therapies.

**Conclusion**: In this nationally comprehensive cohort of MS patients diagnosed in Scotland within the past 10 years, we observed similar rates of PCR-confirmed SARS-CoV-2 infection compared to the general Scottish population, but a small number of excess COVID-19 related deaths. These deaths occurred in individuals with advanced disability who were not receiving DMTs.

## Introduction

Coronavirus disease 2019 (COVID-19), caused by the severe acute respiratory syndrome coronavirus 2 (SARS-CoV-2), emerged in China in late 2019 and was declared a pandemic by the WHO in mid-March 2020 ^1, 2^. An important aspect of the public health response to COVID-19 has been the accurate identification of individuals at risk of severe COVID-19 outcomes, particularly those at high risk of death.

Multiple sclerosis (MS) affects over two million people worldwide and the impact and mortality of COVID-19 on people with MS is a source of major current concern. People with multiple sclerosis are potentially at risk of severe COVID-19 because they are receiving immunosuppressants, may develop significant disability and commonly have comorbidities. Equally, many individuals with MS are of working age and severe COVID-19 avoidance measures may cause social, mental and financial harm.

During the first wave of the COVID-19 pandemic guidelines were drawn up by national and international neurological and MS societies to advise people with MS of their risk of severe COVID-19 based on theoretical risks posed by immunotherapy, and evidence of respiratory and bulbar failure ^3, 4^.

As the first wave of the pandemic has emerged, case series and registries of patient-reported and physician-reported COVID-19 in people with MS have enabled the identification of risk factors for severe COVID-19 outcomes ^5– 8^. These studies suggest that age, Expanded Disease Severity Score (EDSS) and comorbidities are risk factors for severe COVID-19 ^5– 8^. These studies also suggest that some immunotherapies such as ocrelizumab may confer modest risk for severe COVID-19 outcomes, but most immunotherapies do not ^7^. Such studies are important for identifying characteristics of affected individuals but rely on spontaneous reporting. There is a need to understand the impact of the pandemic in a representative cohort where cases are ascertained in an unbiased manner, with a focus on severe outcomes and death.

The Scottish MS Register (SMSR) is an NHS Scotland audit tool which has collected data on over 4500 newly diagnosed individuals with MS in Scotland since 2010 ^9^. MS is a lifelong disease, with a clinical course typically lasting over 30 years. Therefore, the Scottish MS register is a nationally comprehensive, incident cohort of people with relatively early MS, in the first decade of disease ^9^. The SMSR can be linked to other healthcare databases across Scotland including infections and deaths.

In light of the recent COVID-19 pandemic, we linked data from the Scottish Multiple Sclerosis Register with the Electronic Communication of Surveillance in Scotland (ECOSS), a Scotland-wide surveillance tool for monitoring infections that are of clinical or public health importance. This flagging system allows neurologists across Scotland to be informed when people with MS under their care develop COVID-19, based on a positive nasopharyngeal PCR test. In addition, linkage to Scottish death records from National Records Scotland was also performed during the period of the pandemic first wave.

In this manuscript we report the findings from this surveillance system during the first wave of the COVID-19 pandemic in Scotland over the period 1 ^st^ March – 31 ^st^ July 2020.

## Methods

### Scottish MS Register: ethics and data governance

The research and governance framework of the SMSR has been previously described ^9, 10^. The Scottish MS Register is an established Scottish national NHS audit, and as such does not require research ethics approval. The aim of the register, which was established in 2010, is to improve the NHS care of people with MS in Scotland. The aims, objectives, permissions and data governance of the SMSR are available from the Register site.

Patient information about how the data is collected and used is provided in the Patient Information Sheet.

### Linkage to ECOSS and mortality databases

Following internal governance review, the ECOSS and SMSR databases were electronically linked in real-time, permitting patients appearing on both databases to be identified weekly using the Scottish national patient unique identifier system (the Community Health Identification number) starting March 1 ^st^ 2020. Local neurologists were informed by the SMSR when a patient under their care had developed PCR confirmed SARS-CoV-2 infection, or had died. This information was used in the routine clinical care of the patient and neurologists fed back deidentified data to the SMSR. Deaths in the absence of a positive COVID-19 PCR results were determined to be COVID-19 related in the clinical judgement of the local clinical neurologist. Collated data was reviewed periodically during the pandemic.

### Comparison of SMSR data with general Scottish population

The population structure of the SMSR was taken from https://www.msr.scot.nhs.uk/Reports/Dashboard-2020.html and was cross-referenced with national death records to exclude individuals who died prior to 1st March 2020.

National background rates of COVID-19, including mortality data, were identified from published PHS datasets. These age and sex-specific rates were used to estimate expected number of positive tests and deaths within the SMSR. COVID-19 test data were taken from the Public Health Scotland Weekly COVID-19 report dated August 2 and included the period 1 ^st^ March – 31 ^st^ July 2020 ^11^. This contains information on all positive and negative nasopharyngeal COVID-19 tests carried through NHS Scotland laboratories and includes results from hospitals, GP practices, drive-through centres, mobile units, and home testing kits. Data on COVID-19 related deaths from 1 ^st^ March to 31 ^st^ July 2020 were obtained from the National Records of Scotland, defined as deaths occurring in any location where COVID-19 was recorded on the death certificate ^12^.

### Statistical analyses

Statistical analyses were performed in R version 3.6.3 using package epiR version 1.0-15. Confidence intervals were approximated using the method of Rothman and Greenland assuming observed events to be Poisson variates and expected events invariate. Under the same assumptions, p values (H0: ratio of observed to expected = 1), were calculated by chi-squared test. Exact confidence intervals and hypothesis testing, where expected counts were low (≤5), were calculated using the mid-P exact method. For reporting statistical significance, the threshold (α) was set at 0.05.

## Results

### (i) PCR-confirmed SARS-CoV-2 cases within the SMSR

**Figure 1.**
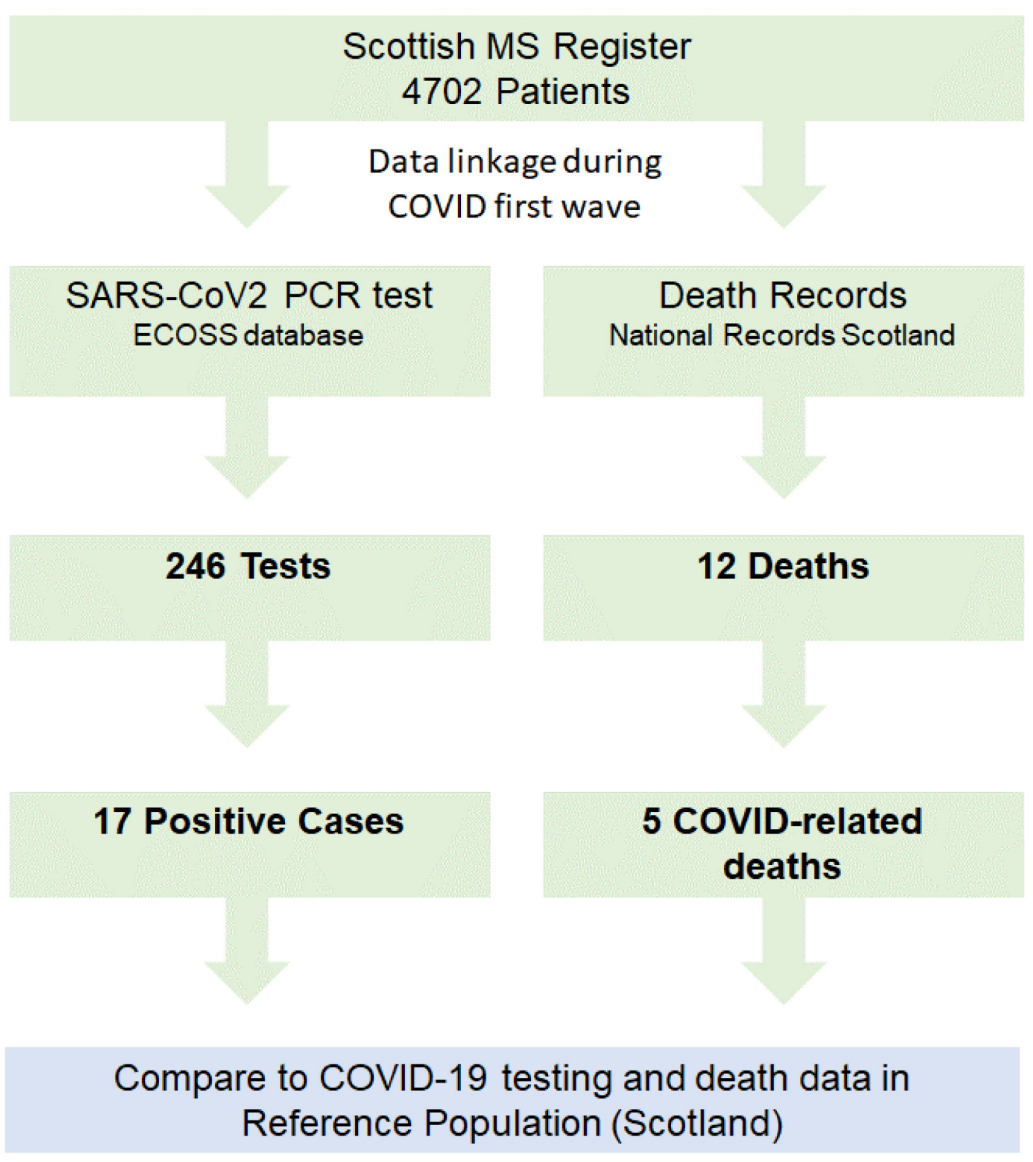
Overview of Scottish Multiple Sclerosis (MS) Register coronavirus disease 2019 (COVID-19) surveillance system. SARS-CoV2: severe acute respiratory syndrome coronavirus disease 2019, ECOSS: Electronic Communication of Surveillance in Scotland.

Of the 4702 people diagnosed with MS since 2010 on the SMSR, 246 (5.2%) underwent SARS-CoV-2 PCR testing during the first wave. Over the same period, 6.7% of the Scottish population underwent testing. Of the 246 SARS-CoV-2 PCR tests carried out in the SMSR population, 17 (6.9%) were positive ( Figure 1). The number of SARS-CoV-2 PCR-confirmed tests we observed in the SMSR was similar to the number expected, based on Scotland-wide testing data. ( Table 1, observed =17, expected = 17.5 (O/E = 0.97, 95% CI: 0.60 – 1.56), X ^2^(d.f. = 1) = 0.014, *p*=0.90).

**Table 1.** Number of PCR-confirmed SARS-CoV2 positive cases in Scottish MS Register March 1 ^st^ 2020 – 31 ^st^ July 2020, compared to Scotland-wide SARS-CoV-2 positive PCR test results.

Sex	Age	SMSR population	Positive COVID-19 tests in Scotland (per 1000 population)	Expected positive COVID-19 tests in SMSR	Observed positive COVID-19 tests in SMSR	O/E (95% CI)
**Female**	**15-44**	1524	3.54	13.8	13	
	**45-64**	1494	4.89			
	**65-84**	261	4.25			
**Male**	**15-44**	647	1.59	3.7	4	
	**45-64**	659	3.14			
	**65-84**	116	5.06			
**Total**				**17.5**	**17**	0.97 (0.60 – 1.56) *p*=0.90

MS: Multiple Sclerosis, SARS-COV-2: Severe acute respiratory syndrome coronavirus 2, SMSR: Scottish MS Register, COVID-19: coronavirus disease 2019, O/E: observed/expected

### (ii) COVID-19-associated deaths within the SMSR

**Figure 2.**
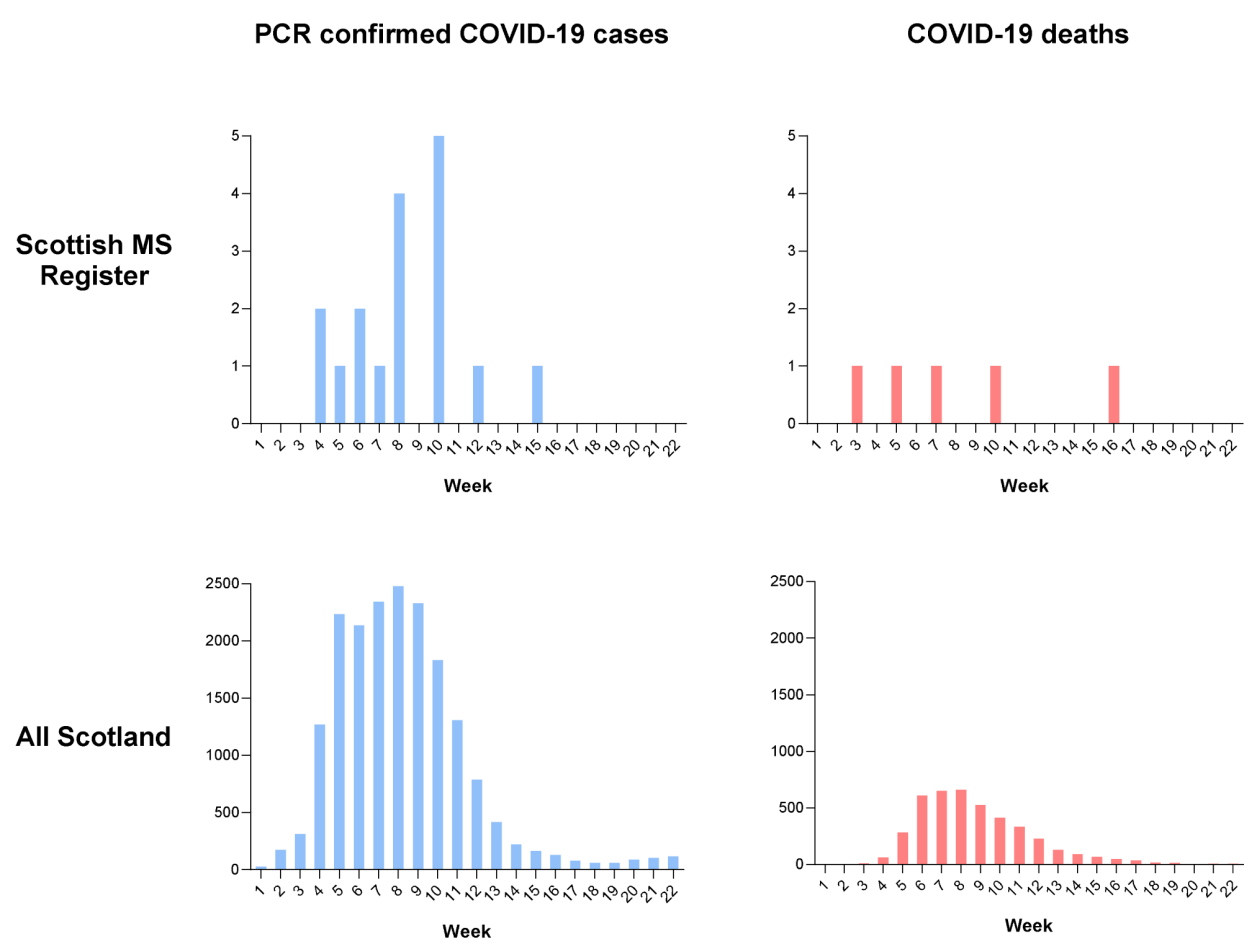
PCR-confirmed severe acute respiratory syndrome coronavirus 2 (SARS-CoV-2) cases and coronavirus disease 2019 (COVID-19) related deaths during the first pandemic wave. PCR-confirmed SARS-CoV2 cases (blue) and COVID-19-related deaths (red) within the Scottish Multiple Sclerosis (MS) Register (top) and Scottish population (bottom). Cases are described in weeks, beginning 1 ^st^ March 2020 (week 1) and ending the week 26 ^th^ July 2020.

Given that the proportion of PCR-confirmed cases were similar between the SMSR and the general population, we next asked whether there were differences in COVID-19 related mortality. During the first pandemic wave 12 deaths of individuals on the SMSR were recorded, 5 of which were identified to be COVID-19 related. This observed number of COVID-19-related deaths in the SMSR cohort is higher than expected, based on COVID-19 related death rate in the general Scottish population over the same time period. ( Table 2, observed COVID-19 related deaths = 5, expected COVID-19 related deaths 1.2, O/E = 4.03, 95% CI = 1.48 – 8.94, *p*=0.01 (mid-P exact)). One individual died after positive PCR-confirmation, and 4 died of clinically suspected COVID-19 but were not tested. The timings of these deaths relative to the wave of COVID-19 attributable deaths in Scotland are shown in Figure 2.

**Table 2.** Number of COVID-19 related deaths in Scottish MS Register March 1 ^st^ 2020 – 31 ^st^ July 2020, compared to Scotland-wide results.

Sex	Age	SMSR population	COVID-19 deaths in Scotland (per 1000 population)	Expected COVID-19 deaths in SMSR ^†^	Observed COVID-19 related deaths in SMSR	O/E (95% CI)
**Female**	**15-44**	1524	0.01	0.71	2	
	**45-64**	1494	0.16			
	**65-84**	261	1.76			
**Male**	**15-44**	647	0.01	0.52	3	
	**45-64**	659	0.31			
	**65-84**	116	2.70			
**Total**				**1.24**	**5**	4.03 (1.48 – 8.94) *p*=0.01

^**†**^Unrounded values sum to total. MS: Multiple Sclerosis, SMSR: Scottish MS Register, COVID-19: coronavirus disease 2019, O/E: observed/expected

In light of the observed excess of COVID-19-related deaths over and above the general population we collated clinical information fed back to the SMSR about COVID-19 attributable deaths. Within the SMSR cohort, all five patients who died due to COVID-19 had advanced disability (EDSS≥7) and none were receiving disease modifying therapies.

## Discussion

Using the Scottish MS Register COVID-19 surveillance system, we found that the proportion of SMSR with PCR-confirmed SARS-CoV2 infection was similar to that of the general Scottish population. However, we observed 5 deaths linked to COVID-19, when ~1 death was expected, based on the Scottish reference population. These deaths all occurred in patients with advanced disability and no patients receiving immunotherapy died.

The SMSR was established in 2010 to capture all incident MS cases in Scotland. The register therefore has the advantage of being nationally comprehensive and captures data from individuals who are in the first decade of the disease, who will have lower disability scores, and higher immunotherapy treatment rates than the prevalent population ^13^.

Our data provide particular insight into the impact of COVID-19 on mortality within the SMSR and suggest that this burden is falling upon patients with advanced disability. However, our analysis has a number of important limitations. Firstly, during the majority of the time period covered by this analysis, SARS-CoV2 PCR swab testing was restricted to a hospital setting or healthcare workers. Throughout the first wave of COVID-19 infection the availability of SARS-CoV2 PCR nasopharyngeal swab tests changed, as did the threshold for testing: initially only hospitalised patients were eligible for tests, followed by healthcare professionals, and finally widespread community testing was introduced. Therefore, many mild cases occurred in the community without PCR confirmation and would not be identified using this surveillance system. Secondly, while the ECOSS database is comprehensive, we cannot be sure that all cases are captured through record linkage, such as those having privately organised tests or not being tested at all. Thirdly, the data linkage described occurs within Scotland only and will not capture COVID-19 diagnoses or deaths outside Scotland. Finally, since the SMSR is primarily an audit tool, detailed clinical data such as severity scores and immunotherapy details are not currently collected across the SMSR.

Caution is also needed in interpretation of our observed versus expected analyses for COVID-19-related deaths because of slight differences in ascertainment between SMSR and National Records Scotland. In the general Scottish population, a COVID-19-related death is defined by National Records of Scotland as a death which records COVID-19 on the death certificate. In the SMSR population, a death was recorded as COVID-19-related by the neurologist.

These results are consistent with an emerging body of evidence derived from other cohort and registry studies which suggest that the burden of severe COVID-19 is falling on individuals with advanced disability rather than those receiving immunotherapies ^5, 6^. Our data do not permit quantification of the risk of death with individual immunotherapies since the SMSR holds only very limited data on the current DMT usage of the population, although over 60% of newly diagnosed patients in the SMSR are offered a DMT at the point of diagnosis ^9^. While it is reassuring that no patients on DMTs in the SMSR cohort died of COVID-19 it is important to bear in mind that UK Government and Scottish Government advice was for all patients with MS to perform stringent social distancing during much of the period captured here. 

These results may be relevant to informing the optimisation of COVID-19 avoidance measures for people with MS in the event of future pandemic waves. In Scotland during the first wave of the pandemic, all patients with MS were advised to perform particularly stringent social distancing. In addition, people with MS who (i) had recently received treatment with alemtuzumab or cladribine or (ii) experienced bulbar or respiratory dysfunction, were advised to adopt even more stringent “shielding” measures. Our analysis is not intended to evaluate the complex risk-benefits outcomes of such interventions. However, it is clear that, within the SMSR cohort, those with advanced neurological disability are a particularly vulnerable population.

## Conclusion

The number of confirmed SARS-CoV-2 infections in the SMSR were observed at a similar frequency to the general population. However, we observed a small number of excess deaths due to COVID-19 in people with MS compared to the general population. These deaths occurred in individuals with advanced disability who were not receiving immunotherapy. These results may help identify people with MS who are vulnerable to severe/fatal COVID-19.

## Data availability

### Source data

#### COVID-19 data

Scotland-wide COVID-19 PCR test data are available online from Public Health Scotland ( https://publichealthscotland.scot/). The specific dataset used is the “Public Health Scotland Weekly Covid-19 Report August 9” (Supplementary Excel File, Tab = Positive AgeSexSIMD); https://beta.isdscotland.org/find-publications-and-data/population-health/Covid-19/Covid-19-statistical-report/. [Accessed: 16-Aug-2020]).

Scotland-wide COVID-19 related mortality data are available online from the National Records of Scotland ( https://www.nrscotland.gov.uk/). The specific dataset used is the “Deaths involving coronavirus (COVID-19) in Scotland Week 31”; https://www.nrscotland.gov.uk/files/statistics/covid19/covid-deaths-report-week-31.pdf. [Accessed: 16-Aug-2020].

### Scottish MS Register

This dataset is held within Public Health Scotland (PHS). All personal details on the register are stored in accordance with Information Services Division (ISD) Guidelines. The most recent Scottish MS Register report and publicly available data are available from: https://www.msr.scot.nhs.uk/Reports/Dashboard-2020.html. Full details of data governance can be found here: https://www.msr.scot.nhs.uk/data.html

The information is collected by the hospital and is collated by the Information Services Division (ISD) at NHS Scotland. ISD has well established systems to protect the privacy of data held on patients and staff. The General Data Protection Regulation (GDPR), the Data Protection Act 2018 and Public Benefit and Privacy Panel for Health & Social Care (PBPP) Guidelines. Application for access to data from the SMSR can be made to nss.isdscottishmsregister@nhs.net. No identifiable information will be passed to any individual or organisation outwith the National Health Service.

## References

[1] World Health Organization: Statement on the second meeting of the International Health Regulations (2005) Emergency Committee regarding the outbreak of novel coronavirus (2019-nCoV). 2020 Reference Source

[2] World Health Organization: World Health Organization COVID-19 Timeline.[Accessed: 15-Sep-2020]. Reference Source

[3] Coles A MS Advisory Group: ABN guidance on the use of disease-modifying therapies in Multiple Sclerosis in response to the COVID19 pandemic. 2020 [Accessed: 03-Aug-2020]. Reference Source

[4] MS International Federation: The coronavirus and MS - updated global advice. 2020 [Accessed: 15-Sep-2020]. Reference Source

[5] Louapre C Collongues N Stankoff B: Clinical Characteristics and Outcomes in Patients With Coronavirus Disease 2019 and Multiple Sclerosis. *JAMA Neurol.* 2020;77(9):1079–1088. 10.1001/jamaneurol.2020.2581 32589189 PMC7320356

[6] Sormani MP Italian Study Group on COVID-19 infection in multiple sclerosis: An Italian programme for COVID-19 infection in multiple sclerosis. *Lancet Neurol.* 2020;19(6):481–482. 10.1016/S1474-4422(20)30147-2 32359409 PMC7191287

[7] Sormani MP De Rossi N Schiavetti I: Disease Modifying Therapies and COVID-19 Severity in Multiple Sclerosis. 2020 10.2139/ssrn.3631244

[8] Evangelou N Garjani A dasNair R: Self-diagnosed COVID-19 in people with multiple sclerosis : a community-based cohort of the UK MS Register. *J Neurol Neurosurg Psychiatry.* 2020; jnnp-2020-324449. 10.1136/jnnp-2020-324449 32855290 PMC7803896

[9] Public Health Scotland: Scottish Multiple Sclerosis Register National Report 2020. 2020 Reference Source

[10] Kearns PKA Paton M O'Neill M: Regional variation in the incidence rate and sex ratio of multiple sclerosis in Scotland 2010-2017: findings from the Scottish Multiple Sclerosis Register. *J Neurol.* 2019;266(10):2376–2386. 10.1007/s00415-019-09413-x 31187189 PMC6765473

[11] Public Health Scotland: Public Health Scotland Weekly Covid-19 Report August 9 (Supplementary Excel File, Tab = Positive AgeSexSIMD).[Accessed: 16-Aug-2020]. Reference Source

[12] National Records of Scotland: Deaths involving coronavirus (COVID-19) in Scotland Week 31. 2020; [Accessed: 16-Aug-2020]. Reference Source

[13] Mackenzie IS Morant SV Bloomfield GA: Incidence and prevalence of multiple sclerosis in the UK 1990-2010: A descriptive study in the General Practice Research Database. *J Neurol Neurosurg Psychiatry.* 2014;85(1):79–84. 10.1136/jnnp-2013-305450 24052635 PMC3888639

