# Supplementary material for: Impact of the first COVID-19 pandemic wave on the Scottish Multiple Sclerosis Register population
Source: Wellcome Open Res. 2020 Nov 25;5:276. [Version 1] doi: 10.12688/wellcomeopenres.16349.1 (PMC7848856; doi:10.12688/wellcomeopenres.16349.1)
Supplement: Peer review history

# Peer review: Reviewer response for version 1

**DOI:** 10.21956/wellcomeopenres.17978.r41940

**Published:** 2021-02-04

**Authors:** Joanne Louise Jones

**Affiliations:** 1 Department of Clinical Neurosciences, University of Cambridge, Cambridge, UK

This very clearly written report describes rates of Covid infection, and Covid-related mortality, in individuals with multiple sclerosis (MS) on the Scottish MS Register (SMSR) during the first wave of Covid infections (March 2020-July 2020). 

 The study concludes that individuals on the SMSR had similar rates of PCR-confirmed SARS-CoV2 infections as the general population. But that there was a small number of excess Covid-related deaths (n=5), all of which occurred in individuals with advanced disease and high levels of disability (EDSS >7.0), who were not receiving DMTs. These findings agree well with other reports. 

 The main strength of this study is the unique SMSR resource, and how this was linked by the research team to an electronic surveillance system for SARS-CoV2 testing results, as well as deaths reported to the National records registry. 

 As pointed out by the authors, the study has a number of limitations (most of which are beyond the control of the research team), including:

1. Limited PCR testing during the first wave (meaning that mild community cases would not have been identified) and the fact that 4 out of the 5 Covid-related deaths were diagnosed clinically. It is also unclear to me why Covid-relatedness for the SMSR cohort was determined by a neurologist rather than by the death certificate (as would be the case for the general population). This may have introduced bias. 
2. Limited clinical details held by the SMSR (which is primarily an audit tool) - for example regarding use of DMTs, co-morbidities and level of disability. Given this, some caution must be applied when extrapolating these data to other clinical groups. 
3. It would also have been interesting for the authors to speculate on how these data may have been influenced by the initial shielding guidance given to individuals with MS. For example, I am unsure if the authors have access to information regarding what proportion of the SMSR cohort would have been advised to shield. But if significant, the risks calculated in this manuscript may be an underestimate. It would also be interesting to know what proportion of patients with advanced disease were living in a care-home setting. 

In conclusion, this is a very interesting paper that makes excellent use of a unique clinical resource (SMSR). It adds further weight to the argument that for further waves/future pandemics greater emphasis should be placed on protecting individuals with advanced MS and higher levels of disability.

Is the work clearly and accurately presented and does it cite the current literature?

Yes

If applicable, is the statistical analysis and its interpretation appropriate?

Yes

Are all the source data underlying the results available to ensure full reproducibility?

Yes

Is the study design appropriate and is the work technically sound?

Yes

Are the conclusions drawn adequately supported by the results?

Yes

Are sufficient details of methods and analysis provided to allow replication by others?

Yes

Reviewer Expertise:

Immunology, T-regulatory cell biology, Tissue Immunity, Multiple Sclerosis, Autoimmunity, Clinical Trials.

I confirm that I have read this submission and believe that I have an appropriate level of expertise to confirm that it is of an acceptable scientific standard.

# Peer review: Reviewer response for version 1

**DOI:** 10.21956/wellcomeopenres.17978.r42356

**Published:** 2021-01-29

**Authors:** Neil Basu

**Affiliations:** 1 Institute of Infection, Immunity & Inflammation, University of Glasgow, Glasgow, UK

A national epidemiological survey which aims to estimate the risk of COVID susceptibility and death in Multiple Sclerosis (MS) employing a national registry of early MS as its population frame.

 Scotland enjoys, almost internationally, unique health care system data linkage capacities. The joined up nature of their health care system and almost complete population coverage enables rapid comprehensive studies such as this. Its also heartening to see how these facilities can be used to immediately inform patient care as evidenced by the flagging system documented in this manuscript.

 The analysis is a useful contribution which provides data which aligns with earlier, less comprehensive reports in this space.

 With the rapid evolution of discovery in this space, it would be helpful for these finding to be contextualized with the most up-to-date extant literature.

 More details regrading the clinical characteristics of the MS registry would be helpful to understand the prevalence of known COVID risk factors. Further, details of what proportion of patients fall into the UK government's extreme social isolation category would help assess whether the calculated risk is an underestimate. 

 Finally, given the known geographical risk factors for both MS and COVID (which conceivably could conflict), a future effort to match controls according to post code (a feasible undertaking) would enhance confidence around the risk estimates.

Is the work clearly and accurately presented and does it cite the current literature?

Partly

If applicable, is the statistical analysis and its interpretation appropriate?

Yes

Are all the source data underlying the results available to ensure full reproducibility?

No

Is the study design appropriate and is the work technically sound?

Yes

Are the conclusions drawn adequately supported by the results?

Yes

Are sufficient details of methods and analysis provided to allow replication by others?

Yes

Reviewer Expertise:

Epidemiology

I confirm that I have read this submission and believe that I have an appropriate level of expertise to confirm that it is of an acceptable scientific standard.

# Peer review: Reviewer response for version 1

**DOI:** 10.21956/wellcomeopenres.17978.r41595

**Published:** 2020-12-18

**Authors:** Fredrik Piehl

**Affiliations:** 1 Department of Clinical Neuroscience, Karolinska Institutet, Stockholm, Sweden

In this paper, Fernandes and co-workers report outcomes with Covid-19 in a Scottish MS population. A great deal of attention has been given to the possible impact of disease-modulatory treatments (DMT) for MS on COVID-19 outcomes. However, most existing data comes from spontaneous reports, constituting a limitation for extrapolating results on a population level. In this study, the Scottish MS registry (SMSR) was linked to an electronic surveillance system for Sars-CoV2 testing results, as well as deaths reported to the National Records registry. Top line results comprise a similar rate of positive Sars-CoV2 testing results as the general population, but increased mortality compared to age and sex matched controls. All five deaths reported occurred in individuals with a high disability level who were not currently treated with DMT. The authors correctly mention certain limitations with the study, such as that a relatively small proportion of individuals were tested for Sars-CoV2 with PCR, the reporting system for test results not covering testing in private clinics, the fact that four out of five deaths connected to Covid-19 were clinically diagnosed and that the SMSR does not include details on DMT and severity scores. In particular, without knowledge about disability levels and DMT status, caution must be exerted before extrapolating results to other populations that may be structured in a different way. In addition, the SMSR includes individuals being diagnosed with MS in the last decade. It is unclear to this reviewer if the coverage has been validated by some means. Also, the age structure of the reported population is quite high given the disease duration, likely reflecting a delay between disease onset and diagnosis. Only age and sex are reported for the five identified deaths, and other factors, such as co-morbidity and other concomitant treatments apart from DMTs, may also have been of relevance. Nevertheless, population-based studies, such as this, are still rare and the presentation of results, statistical methods and resulting conclusions are relevant given the available data.

Is the work clearly and accurately presented and does it cite the current literature?

Yes

If applicable, is the statistical analysis and its interpretation appropriate?

Yes

Are all the source data underlying the results available to ensure full reproducibility?

Yes

Is the study design appropriate and is the work technically sound?

Yes

Are the conclusions drawn adequately supported by the results?

Yes

Are sufficient details of methods and analysis provided to allow replication by others?

Yes

Reviewer Expertise:

multiple sclerosis, clinical and experimental aspects, including treatments

I confirm that I have read this submission and believe that I have an appropriate level of expertise to confirm that it is of an acceptable scientific standard.
